# Supplementary material for: Health Literacy in Early Childhood: A Systematic Review of Empirical Studies
Source: Children (Basel). 2022 Jul 28;9(8):1131. doi: 10.3390/children9081131 (PMC9406303; doi:10.3390/children9081131)
Supplement: Supplementary file 1 [file children-09-01131-s001.zip › children-1810873-supplementary.pdf]

Table S1: Newcastle-Ottawa Quality Assessment Scale for cross-sectional quantitative studies

Author (Year)	Study design	Selection				Comparability	Outcome	
		Representativeness of the sample	Sample size	Non-respondents	Ascertainment of the exposure (risk factor)	Based on design and analyses	Assessment of the outcome	Statistical test
**Tabacchi et al. (2019) [49]**	cross-sectional, quantitative	*	*	*	**	*		*
**Tabacchi et al. (2021) [51]**	cross-sectional, quantitative	*	*	*	*	*	**	*
**Tsakpounidou et al. (2021) [52]**	cross-sectional, quantitative				*	*	**	

NEWCASTLE - OTTAWA QUALITY ASSESSMENT SCALE (adapted for cross sectional studies)

Selection: (Maximum 5 stars)

1) Representativeness of the sample:

a) Truly representative of the average in the target population. * (all subjects or random sampling)

b) Somewhat representative of the average in the target population. * (nonrandom sampling)

c) Selected group of users.

d) No description of the sampling strategy.

2) Sample size:

a) Justified and satisfactory. *

b) Not justified.

3) Non-respondents:

a) Comparability between respondents and non-respondents characteristics is established, and the response rate is satisfactory. *

b) The response rate is unsatisfactory, or the comparability between respondents and non-respondents is unsatisfactory.

c) No description of the response rate or the characteristics of the responders and the non-responders.

4) Ascertainment of the exposure (risk factor):

a) Validated measurement tool. **

b) Non-validated measurement tool, but the tool is available or described.*

c) No description of the measurement tool.

Comparability: (Maximum 2 stars)

1) The subjects in different outcome groups are comparable, based on the study design or analysis. Confounding factors are controlled.

a) The study controls for the most important factor (select one). *

b) The study control for any additional factor. *

Outcome: (Maximum 3 stars)

1) Assessment of the outcome:

a) Independent blind assessment. **

b) Record linkage. **

c) Self report. *

d) No description.

2) Statistical test:

a) The statistical test used to analyze the data is clearly described and appropriate, and the measurement of the association is presented, including confidence intervals and the probability level (p value). *

b) The statistical test is not appropriate, not described or incomplete.

This scale has been adapted from the Newcastle-Ottawa Quality Assessment Scale for cohort studies to perform a quality assessment of cross-sectional studies for the systematic review, “Panethnic Differences in Blood Pressure in Europe: A Systematic Review and MetaAnalysis”. [57]

Table S2: Quality assessment for cross-sectional qualitative studies

Author (Year)	Study design	Abstract and title	Introduction and aims	Method and data	Sampling	Data analysis	Ethics and bias	Results	Transferability or generalisability	Implications and usefulness	Total point	Grade
**Charsley et al. (2018) [44]**	cross-sectional, qualitative	4	3	4	3	4	3	4	4	3	32	A
**Derwig et al. (2021) [45]**	cross-sectional, qualitative	3	4	4	4	4	4	4	4	4	35	A
**Drummond et al. (2013) [46]**	cross-sectional, qualitative	2	3	3	3	3	2	4	3	3	26	B
**Privitera et al. (2015) [47]**	cross-sectional, qualitative	3	3	4	4	4	4	4	4	4	34	A
**Stålberg et al. (2016) [48]**	cross-sectional, qualitative	4	4	4	4	4	4	4	4	4	36	A

4: good, 3: fair, 2:poor, 1: very poor
high quality (A): 30–36 points; medium quality (B): 24–29 points; low quality (C): 9–24 points.

The questions are as follows:

1. Abstract and title. Did they provide a clear description of the study?

   Good: structured abstract with full information and clear title. Fair: abstract with most of the information. Poor: inadequate abstract. Very poor: no abstract.

2. Introduction and aims. Was there a good background section and clear statement of the aims of the research?

   Good: full but concise background to discussion/study containing up-to-date literature review and highlighting gaps in knowledge; clear statement of aim AND objectives including research questions. Fair: some background and literature review; research questions outlined. Poor: some background but no aim/objectives/questions OR aims/objectives but inadequate background. Very poor: no mention of aims/objectives; no background or literature review.

3. Method and data. Is the method appropriate and clearly explained?

   Good: method is appropriate and described clearly (e.g. questionnaires included); clear details of the data collection and recording. Fair: method appropriate, description could be better; data described. Poor: questionable whether method is appropriate; method described inadequately; little description of data. Very poor: no mention of method AND/OR method inappropriate AND/OR no details of data.

4. Sampling. Was the sampling strategy appropriate to address the aims?

   Good: details (age/gender/race/context) of who was studied and how they were recruited and why this group was targeted; the sample size was justified for the study; response rates shown and explained. Fair: sample size justified; most information given but some missing. Poor: sampling mentioned but few descriptive details. Very poor: no details of sample.

5. Data analysis. Was the description of the data analysis sufficiently rigorous?

   Good: clear description of how analysis was carried out; description of how themes derived/respondent validation or triangulation. Fair: descriptive discussion of analysis. Poor: minimal details about analysis. Very poor: no discussion of analysis.

6. Ethics and bias. Have ethical issues been addressed and has necessary ethical approval been gained?Has the relationship between researchers and participants been adequately considered?

   Good: ethics: when necessary, issues of confidentiality, sensitivity and consent were addressed; bias: researcher was reflexive and/or aware of own bias. Fair: lip service was paid to above (i.e. these issues were acknowledged). Poor: brief mention of issues. Very poor: no mention of issues.

7. Results. Is there a clear statement of the findings?

   Good: findings explicit, easy to understand and in logical progression; tables, if present, are explained in text; results relate directly to aims; sufficient data are presented to support findings. Fair: findings mentioned but more explanation could be given; data presented relate directly to results. Poor: findings presented haphazardly, not explained and do not progress logically from results. Very poor: findings not mentioned or do not relate to aims.

8. Transferability or generalisability. Are the findings of this study transferable (generalisable) to a wider population?

Good: context and setting of the study are described sufficiently to allow comparison with other contexts and settings, plus high score in Q4 (sampling). Fair: some context and setting described but more needed to replicate or compare the study with others, plus fair score or higher in Q4. Poor: minimal description of context/setting. Very poor: no description of context/setting.

9. Implications and usefulness. How important are these findings to policy and practice?

   Good: contributes something new and/or different in terms of understanding/insight or perspective; suggests ideas for further research; suggests implications for policy and/or practice. Fair: two of the above. Poor: only one of the above. Very poor: none of the above. [58]

Table S3: Summary of Risc of Bias in interventional studies

Anwar et al. (2020) [42]	Brega et al. (2016) [43]	Tabacchi et al. (2020) [50]	Zhou et al. (2020) [53]	
+	+	-	?	**Random sequence generation (selection bias)**
-	?	?	?	**Allocation concealment (selection bias)**
?	-	-	+	**Blinding of participants and personnel (performance bias)**
?	-	-	+	**Blinding of outcome assessment (detection bias)**
+	+	+	+	**Incomplete outcome data (attrition bias)**
+	+	+	+	**Selective reporting (reporting bias)**
				**Other sources of bias.**

Red: High risk; Yellow: Unclear risk; Green: Low risk [59]

Table S4: Interventions in the included studies

Author/Year	Intervention group	Control group	The intervention	Input measurement	Duration of intervention	Interventions Outcomes
Anwar et al. (2020) [42]	5-6-year-old children participating in the SIMS Program (Senyuman Indah Milik Semua or "Beautiful Smile" for All') (n=344)	5-6-year-old children involved in regular Preschool Oral Healthcare Program (POHP) (n=309)	SIMS program:<br>1. In-class oral health lessons by teachers using the teacher's oral health education booklet<br>2. In-school daily supervised tooth brushing with fluoride toothpaste<br>3. Supervised home tooth brushing at night by parents/guardians	Oral examination	6 months	The SIMSP was more effective for reducing children's plaque scores and soft drink intake than POHP
Brega AG et al. (2016) [43]	3-5 year-old children in Navajo Head Start program (n=1016)	-	Caregivers applicate fluoride varnish and made education during 2 years. Fluoride varnish and oral health education were given four times per school year to children. Parents received education 3 times every year. Participants received toothbrushes and toothpaste for all family members.	The BRFQ contains parent and child characteristics; oral health knowledge, attitudes, behaviour and outcomes.	2-year	Children's health behaviour and HL were improved during the study period.
Tabacchi et al. (2020) [50]	An intervention subgroup (100 children of the participant) was used judging the discriminant validity of the preschool-FLAT tool.	The Control group was used to judge the discriminant validity of the preschool-FLAT tool (n=27)	Oral sessions and activities: each session of the food laboratory consisted of a brief oral session, using the story-telling methodology, subsequently, practical sessions were conducted.<br><br>Module 1: body weight and foods<br>Module 2: food quality/quantity and health		A total of ten hours during four months (around twenty minutes/session)	The intervention is supposed to be effective in increasing performances and cognitive abilities in preschoolers. The preschool-FLAT represents good psychometric properties, adequate validity and internal consistency.

			Module 3: eat organic and follow seasons!<br>Module 4: Let's know traditional Sicilian foods<br>Module 5: Let's build the food pyramid			
Zhou et al. (2020) [53]	Intervention group 1: preschool children with autism (n=87)<br><br>Intervention group 2: preschool children with special needs without autism (n=94)	-	A social story-assisted toothbrushing training was provided to all the participants by a dental assistant.<br>After that, the child was encouraged to brush their own teeth in front of a mirror<br>After toothbrushing training, feedback were given to parents, based on children's performance.<br><br>Parents were encouraged to read the social stories to their children before or during daily toothbrushing once per day	Children's tooth brushing performance, oral hygiene status, and gingival health status were assessed at baseline	6 months	Both intervention groups showed similar improvement in toothbrushing performance<br><br>Children whose parents perceived that the social story was useful, were more likely to have better gingival health status.

Table S5: Content of the studies

Author/Year	Study Aim	Study population	Methods	Research tools	Results	Conclusion
Anwar et al. (2020) [42]	To compare the effect of SIMSP (Senyuman Indah Milik Semua' or 'Beautiful Smile for All') Programme to the POHP (Preschool Oral Healthcare Programme) on preschool children's oral health parameters	5-6 years old children (n=653)	pragmatic, cluster-randomized, parallel-group, matched pair, controlled trial	Oral examination; Oral health education booklet	SIMSP was effective in reducing children's plaque scores and soft drink intake compared to the POHP over 6 months.	The study contributes knowledge of the importance of effective tooth brushing, OHE by teachers, and parents' support for children's oral health.<br>The adoption of the SIMS program into the National Preschool Oral Healthcare Program (POHP) can improve the oral health literacy of children.
Brega AG et al. (2016) [43]	The aim of the study was to examine the association of parental health literacy with oral health knowledge and attitudes. Also to measure the adherence to recommended oral health behavior and indicators of oral health.	3–5 years old children and their parents (n=1016)	A 2-year period follow-up study	Basic Research Factors Questionnaire (BRFQ)	Parents had a mean health literacy score, Oral health knowledge scores also were strong. Adherence to recommended oral health behaviors was low, and parents' oral health attitudes were positive. Health literacy was significantly associated with knowledge and behavior, Parents with a better HL level have better adherence to good oral health behavior.	Parental health literacy is associated with oral health attitudes, behavior, and both parental and pediatric outcomes.
Charsley et al. (2018) [44]	To investigate young children's perception of fatness.	4-7 years old children (n=85)	Face-to-face study: qualitative; children's drawing	Picture based test: Character drawings; Character assessment; Body size rating	From children's perspective, obesity is not the most significant feature of a character. Children failed to see the fat characters as fat. Children spoke about the health impact of fatness.<br>During friendship choices, children preferred a friend who was a healthy weight over one who was fat.	Understanding young children's attitudes, and knowledge about obesity is important.<br>The challenge is how to better inform children about obesity and healthy weight.
Derwig et al. (2021) [45]	To explore the children's experiences and attitudes related to health promotion	4 years old children (n=16)	Projective test: thematic structured interviews with children based on	Picture based test: Illustrations with suggested questions about the everyday activities (nutrition,	The children:<br>(1) like to be actively involved in health care situations.<br>(2) are able to be active and reflective in interpreting health messages.	Children from a young age can take an active role in their health and are able to process health information.

			pictures, (average: 36 min; range 11-50 min) related to health messages: promoting good nutrition, sufficient physical activity, healthy teeth and quality sleep	sleeping, toothbrushing, physical activity)	(3) were health-conscious and recognized basic health concepts.	
Drummond et al. (2013) [46]	To explore how boys view food in their lives particularly through a health oriented and gendered lens.	5-10 years old children (n=33)	longitudinal qualitative research: face to face study: focus group interview; projective test;	5-6 years old boys were asked to draw pictures of aspects associated with sport, health and physical activity. Picture-based test.	Boys perceived the concept of health to be inextricably linked to food and nutrition, more so than any other factor. Boys have developed a perception of the role of gender in portion size and food choice during early childhood.	The role of a more holistic approach to health promotion and health-promoting behaviours is a highlight issue in early childhood. Results can help develop strategies to assist boys in making healthy food selection, which will support their food-related health literacy.
Privitera et al. (2015) [47]	To test the utility of the image-based labeling strategy to promote healthy food choices among children.	5-11 years old children (n=64, including 28 children aged 5-8)	laboratory study: observing food choice decisions through "Emo-labeling" image-based labeling strategy	observation protocol according to children's food choice decisions	Children chose more healthy options at most grade levels when the emo labels were present. First graders showed the largest increase in healthy food choices across all grade levels with emo labels added.	"Emo-labeling" was effective at increasing healthy food choices
Stålberg et al. (2016) [48]	To describe how younger children perceive being in a health-care situation.	3-5 years old children (n= 43)	Face-to-face study: Semi-structured interviews; drawing about experiences; projective test	Interview questions; Picture-based test: vignettes showing health-care situations accompanied by short stories; Drawing about experiences in a health-care situations;	Children view themselves as important actors in a healthcare situation. Children would like to be informed directly by professionals, with language appropriate to their level.	The children's perceptions enable professionals to create a mutual understanding which will contribute to the increased involvement of children and to improving the level of the children's health literacy.

Tabacchi et al. (2019) [49]	To investigate the food literacy level in preschoolers;<br>To evaluate the effect of potential predictors and the associations with gross motor and emergent literacy skills.	3-6 years old children (n=921)	Cross-sectional study (measurements of physical condition and skills)	Anthropometric measurements (body weight, height);<br>'Preschool-FLAT' (Food Literacy Assessment Tool);<br>The Italian version of the gross motor development test and the PRCR-2/2009;<br>The applied research tool uses pictures, activities and tangible experiences with food and food products.	Children had an overall good knowledge of the main food categories, they were able to recognize and name the healthy and non-healthy foods, discriminate between different portions, and understand the relationship between portion and health.<br>Children understand the relationship between food and environment, the meaning of organic food and food seasonality.<br>The female gender and older age are independent predictors of better food literacy.<br>Emergent literacy skills were significantly associated with FL scores.	Children raised in an environment where both motor and cognitive skills are enhanced can have better chances of increasing FL and success at school.<br>The need for monitoring FL and its predictors since early age is to be emphasized.
Tabacchi et al. (2020) [50]	To assess the validity and internal consistency of the preschool-FLAT (Food Literacy Assessment Tool)	3–6 years old children (n=505)	Oral sessions and activities on food-related aspects	The preschool-FLAT (Food Literacy Assessment Tool)	The intervention is supposed to be effective in increasing performances and cognitive abilities in preschoolers<br>The preschool-FLAT represents good psychometric properties, adequate validity and internal consistency.	Preschool children need to receive both education sessions and practical lab activities in the field of nutrition to take advantage in terms of learning skills.<br>The preschool-FLAT is a kind of measure-tool specifically targeted to 3–6 years old children that could be effectively used to assess food literacy.
Tabacchi et al (2021) [51]	To investigate the extent to which maternal food habits and physical activity (PA) level predict food-related aspects, PA practice and gross-motor development in preschool children attending kindergartens from low socioeconomic (SE) level	3-6 years old children and their mothers (n=79) (disadvantaged families)	cross-sectional study conducted within the Training-to-Health Project	Questionnaire: items to assess lifestyle and socio-demographic aspects both on mothers and their children;<br>Weight and height of children were measured;	Mothers' food habits are highly associated with children's food habits.<br>A higher mothers' BMI is significantly correlated to incorrect food habits and to higher BMI of their children.<br>No mother perceived her own child as obese while 11.4% were measured and classified as obese; only 2.5% perceived their children as overweight, while 19.0% were measured and classified as overweight.	Children's food habits and food literacy are significantly influenced by mothers' food habits, BMI and education, while mothers' occupation seems not to be an important predictor.

	urban areas and to provide a comprehensive interaction path			Quotient of Gross Motor Development (QGMD); preschool Food Literacy Assessment Tool (FLAT);		
Tsakpounidou et al. (2021) [52]	To examine the level of stroke symptomatology and stroke preparedness knowledge in young children	4-6,5 years old children (n=123)	Face to face study: cross-sectional survey (input measurement before participation in the educational program FAST 112 Heroes)	Picture-based stroke literacy test with verbal explanations	More than half of the sample could recognize stroke symptoms.<br>The children's baseline stroke knowledge is low.<br>Children do not have sufficient knowledge on how to react appropriately in the event of a stroke.	Awareness programs focusing on developing children's stroke literacy are needed
Zhou et al. (2020) [53]	To teach toothbrushing skills to preschool children with special needs	preschool aged children (n=181) (special needs)	Intervention through oral hygiene program and social stories involving dental assistant and parents	Validated toothbrushing social story (the social story was presented by a booklet, and the steps of toothbrushing were demonstrated on each page)	Toothbrushing performance, oral hygiene, and gingival status of the recruited children were significantly improved after using social stories.	Social story intervention could be used to improve toothbrushing skills among children with or without autism, and it could be served as a potential oral health promotion activity for young children.<br>The social story-based health promotion could be implemented among children with special needs.
